# Risk factors of osteoporosis in healthy Moroccan men

**DOI:** 10.1186/1471-2474-11-148

**Published:** 2010-07-05

**Authors:** Abdellah El Maghraoui, Merieme Ghazi, Salim Gassim, Imad Ghozlani, Aziza Mounach, Asmaa Rezqi, Mohamed Dehhaoui

**Affiliations:** 1 Rheumatology Department, Military Hospital Mohammed V, PO Box 1018, Rabat, Morocco; 2 Statistics Department, Agronomic and veterinary institute Hassan II, Rabat, Morocco

## Abstract

**Background:**

Although not as common as in women, osteoporosis remains a significant health care problem in men. Data concerning risk factors of osteoporosis are lacking for the male Moroccan population. The objective of the study was to identify some determinants associated to low bone mineral density in Moroccan men.

**Methods:**

a sample of 592 healthy men aged 20-79 years was recruited from the area of Rabat, the capital of Morocco. Measurements were taken at the lumbar spine and proximal femurs using DXA (Lunar Prodigy Vision, GE). Biometrical, clinical, and lifestyle determinants were collected. Univariate, multivariate, and logistic regression analyses were performed.

**Results:**

the mean (SD) age of the patients was 49 (17.2) years old. The prevalence of osteoporosis and osteopenia were 8.7% and 52.8%, respectively. Lumbar spine and hip BMD correlated significantly with age, weight and BMI. When comparing the subjects according to the WHO classification, significant differences were revealed between the three groups of subjects for age, weight and BMI, prevalence of low calcium intake and low physical activity. The multiple regression analysis found that only age, BMI, and high coffee consumption were independently associated to the osteoporotic status.

**Conclusion:**

ageing and low BMI are the main risk factors associated with osteoporosis in Moroccan men.

## Background

Osteoporosis is characterized by a reduction in bone mineral density (BMD), associated with skeletal fragility, and an increased risk of fracture. Men account for 33-50% of all vertebral fractures, 20-35% of all femoral fractures and 15% of all distal forearm fractures[1,2]. Moreover, fractures in men results in a higher morbidity and mortality than those observed in women [2,3]. A prior fracture seems to confer a higher relative refracture risk (2.8- to 4.3-fold) that yields a similar absolute refracture risk to that of women of the same age with an initial fracture[4]. Preventing the first such fracture may have major public health implications. Thus, understanding the determinants of osteoporosis in men may reduce the burden of disease through facilitating better prevention strategies. In Morocco, the incidence of hip fractures in men has been estimated to 58/100,000 inhabitant over 50[5] and the prevalence of vertebral fractures was estimated to 26% of men over 50[6].

Dual energy x-ray absorptiometry (DXA) is recognized as the reference method to measure BMD accurately and reproducibly[7]. Although BMD should not be used in isolation to predict fractures, BMD predicts osteoporotic fractures in men independently of age, body weight, and prevalent fractures and regardless of the site of measurement[8]. Several studies have tried to identify the main determinants of low BMD in men. However, almost all of the published studies in the literature come from American, European or Asian countries. The World Health Organization (WHO) has developed recently a tool (FRAX) that integrates both clinical risk factors and BMD (femoral neck T-score) in order to predict the 10-year probabilities for hip fracture and major osteoporotic fracture (clinical spine, hip, forearm, or shoulder)[9]. This tool has also been validated in men from some European, American and Asian studies. Thus, data about risk factors of low BMD in men in the south bank of the Mediteranean Sea are lacking. Furthermore, we have already shown some differences and specificities in risk factors of osteoporosis in women in our region compared to western populations[10,11]. Therefore, the aim of this cross-sectional study was to determine risk factors associated to low bone mineral density in Moroccan men.

## Methods

### Subjects

A total of 592 healthy Moroccan men (age range: 20-79 yr) living in the Rabat area participated in the present study. The subjects were volunteers aged between 20 and 79 years. The recruitment was made in part among hospital staff, university students and lay people contacted by word of mouth and through advertisements. We also asked some general practitioners to invite healthy men to participate to the study. Though the sample was not a true probability sample, care was taken to ensure representativeness of the general population, enrolling nearly ten subjects per year of age and with a particular regard to the inclusion of a wide range of body sizes and activities.

The BMD of the lumbar spine and proximal right femur of these male volunteers with no previous history of bone disease was measured after they gave informed consent. The study was approved by the local Ethics Committee. All subjects were fully ambulatory. Screening was done by physical examination and questionnaires. Men using medications affecting calcium metabolism and those with medical conditions known to affect bone metabolism or with a history of major systemic disorder were excluded. Thus, we excluded subjects with non-Caucasian origin, gastrectomy, intestinal resection, recent hyperthyroidism or hyperparathyroidism, treatment with corticosteroids for more than 6 months, or recent severe immobilization. We did not exclude individuals using inhalation steroids or with certain lifestyle habits, such as heavy smoking, being sedentary, being athletic, or having a high or low calcium intake, which are examples of voluntary factors that may have some impact on bone metabolism.

Each subject completed a standardized questionnaire designed to document putative risk factors of osteoporosis. The questionnaire collected information on life style, smoking habits, and level of physical activity in leisure time, along with calcium consumption and the use of vitamins and medications. Height and weight were measured in our centre before DXA measurement with light indoor clothes on, but without shoes. Body mass index (BMI) was calculated by dividing weight in kilograms by height in meters squared. The men were asked whether they usually drank milk, coffee, soft drinks or alcohol; if they ate cheese or yogurt; if they did gymnastics or jogging/walking and if they smoked tobacco. If the answer was positive, they were asked to quantify their average current consumption (evaluated on the 7 day prior to the interview) of milk or yogurt (mL/day), cheese (g/day) and soft drinks, wine and/or spirits (mL/day). Tobacco smoking was quantified as average number of cigarettes (smoked/day) multiplied by the number of years of smoking, gymnastics as min/week, or jogging/walking as min/day. Finally, patients were categorized as never smokers, ex-smokers and current smokers; high, normal and low calcium intake (more than 1500 mg/day, between 800 and 1500 day-1 and below 800 mg/day, respectively); high, moderate and low physical activity (more than 3, 2-3 and below1 hour/week, respectively); high, moderate and low coffee consumption (more than 3, 1-2 and less than 1 cups/day, respectively). The men were also asked about history of "traumatic fractures" regardless of the importance of trauma.

In total, 678 men were screened. Among them 86 individuals were excluded from the study according to predetermined exclusion criteria, whereas 592 met all inclusion criteria and were invited to participate in the BMD measurement. The age range of the subjects was 20-79 years (mean ± SD 49.1 ± 17.2). The age distribution and some other basic parameters are shown in Table 1.

**Table 1:** characteristics of the studied population (*N *= 592).

Variables	
Age: mean (SD), years	49.1 (17.2)
Weight: mean (SD), kgs	73.7 (12.6)
BMI: mean (SD), kg/m^2^	25.0 (3.9)
History of traumatic fractures, n (%)	84 (14.2)
Current smoking, n (%)	93 (15.7)
High coffee consumption, n (%)	191 (32.3)
Low calcium intake, n (%)	310 (52.4)
Low physical activity, n (%)	262 (44.3)
Total hip BMD: mean (SD), g/cm^2^	1.033 (0.16)
Total hip BMD: mean (SD), T-score	-0.71 (1.08)
Femoral neck BMD: mean (SD), g/cm^2^	0.974 (0.17)
Femoral neck BMD: mean (SD), T-score	-0.99 (1.10)
Lumbar spine BMD: mean (SD), g/cm^2^	1.126 (0.16)
Lumbar spine BMD: mean (SD), T-score	-0.50 (1.12)

### BMD Measurement

Bone mineral density was determined by a Lunar Prodigy Vision DXA system (Lunar Corp., Madison, WI). The DXA scans were obtained by standard procedures supplied by the manufacturer for scanning and analysis. All BMD measurements were carried out by 2 experienced technicians. Daily quality control was carried out by measurement of a Lunar phantom. At the time of the study, phantom measurements showed stable results. The phantom precision expressed as the coefficient of variation percentage was 0.08. Moreover, reproducibility has been assessed by the same 2 technicians in clinical practice and showed a smallest detectable difference of 0.04 g/cm2 (spine) and 0.02 (hips)[12]. Patient BMD was measured at the lumbar spine (anteroposterior projection at L1-L4 and L2-L4, but only L2-L4 results were presented to be compared with the published studies, which most of all used this site) and at the femurs (i.e., femoral neck, trochanter, and total hip). The World Health Organization (WHO) classification system was applied, defining osteoporosis as T-score ≤-2.5 and osteopenia as -2.5 < T-score < -1. Study participants were categorized by the lowest T-score of the L1-4 lumbar spine, femur neck, or total femur. The Moroccan male normative database was used for T-score calculation: the mean (SD) values for young normal adults in the Moroccan male normative database were 1.205 g/cm2 (0.15) for lumbar spine, 1.147 g/cm2 (0.16) for femoral neck, and 1.161 g/cm2 (0.16) for total hip[13].

### Statistical Analysis

Results are presented as means (SD) and categorical variables are expressed as frequencies. Associations between continuous variables were examined by Pearson correlation coefficient. Comparison between patients according to the WHO classification system was done using analysis of variance (ANOVA) for quantitative variables and chi-square test for qualitative variables. Stepwise multiple regression analysis was then used to study the determinants of BMD. The level for significance was taken as p ≤ 0.05. Excel 2007 and SPSS 16.0 were used for statistical analysis.

## Results

Characteristics of the population are displayed in Table 1. The mean (SD) age of the patients was 49 (17.2) years old. The prevalence of osteoporosis and osteopenia were 8.7% and 52.8%, respectively. Lumbar spine and hip BMD correlated significantly with age, weight and BMI (Table 2).

**Table 2:** correlation between bone density parameters and age, weight and BMI

		Age (yrs)	Weight (Kg)	BMI	LS BMD (g/cm^2^)	TH BMD (g/cm^2^)	FN BMD (g/cm^2^)
Age (yrs)	r	1	-0.06	0.15**	-0.31**	-0.47**	-0.61**
	p		0.109	<0.0001	<0.0001	<0.0001	<0.0001
Weight (Kg)	r	-0.06	1	0.87**	0.29**	0.32**	0.30**
	p	0.109		<0.0001	<0.0001	<0.0001	<0.0001
BMI (kg/m^2^)	r	0.15**	0.87**	1	0.18**	0.20**	0.12**
	p	<0.0001	<0.0001		<0.0001	<0.0001	0.002
LS BMD (g/cm^2^)	r	-0.31**	0.29**	0.18**	1	0.68**	0.65**
	p	<0.0001	<0.0001	0.000		<0.0001	<0.0001
TH BMD (g/cm^2^)	r	-0.47**	0.32**	0.20**	0.68**	1	0.90**
	p	<0.0001	<0.0001	<0.0001	<0.0001		<0.0001
FN BMD (g/cm^2^)	r	-0.61**	0.30**	0.12**	0.65**	0.90**	1
	p	<0.0001	<0.0001	0.002	<0.0001	<0.0001	

** means correlation is significant at the 0.01 level (2-tailed).

BMI: body mass index; LS: lumbar spine; TH: total hip; FN: femoral neck.

Every anatomical region has a different rate of bone loss. Significant changes were also evident in the lumbar spine (0.3% per year) and femur BMD subregions (% per year): neck (0.6%), trochanter (0.3%), and total hip (0.4%) in males between 20 and 79 years of age, respectively. According to WHO criteria, 52 (8.7%) patients had osteoporosis, 313 (52.8%) osteopenia and 227 (38.3%) normal BMD. When comparing the subjects according to the WHO classification, significant differences were revealed between the three groups of subjects for age, weight and BMI, prevalence of low calcium intake and low physical activity. All the latter characteristics are detailed in Table 3.

**Table 3:** comparison between patients according to WHO classification of osteoporosis.

	No N = 227	Ope N = 313	OP N = 52	p
Age: mean (SD), years	39.1 (14.2)	53.7 (15.6)	65.7 (13.0)	<0.0001
Weight: mean (SD), kgs	77.6 (12.1)	72.3 (12.0)	64.7 (11.3)	<0.0001
BMI: mean (SD), kg/m^2^	25.5 (4.0)	24.9 (3.8)	23.2 (3.8)	0.001
History of traumatic fractures, n (%)	27 (11.9)	45 (14.4)	12 (23.1)	NS
Current smoking, n (%)	31 (13.7)	64 (20.4)	11 (21.2)	NS
High coffee consumption, n (%)	82 (36.1)	92 (29.4)	17 (32.7)	NS
Low calcium intake, n (%)	78 (34.4)	193 (61.7)	39 (75.0)	<0.0001
Low physical activity, n (%)	84 (37.0)	203 (64.9)	43 (82.7)	<0.0001

Comparison used analysis of variance (ANOVA) for quantitative variables and chi-square test for qualitative variables.

BMI: body mass index; Ope: osteopenia; OP: osteoporosis.

After including in a stepwise multiple regression analysis the statistically significant variables cited above, it was found that only age, BMI, and high coffee consumption were independently associated to the osteoporotic status (Table 4). When running the procedure with the total hip T-score, only age and BMI were retained.

**Table 4:** results of multivariate logistic regression analysis for osteoporosis

	Lumbar spine osteoporosis	Total hip osteoporosis	Osteoporosis any site
Age	1.05 [1.02-1.09]*	1.09 [1.05-1.14]*	1.07 [1.03 - 1.10]*
BMI	0.90 [0.80-0.99]*	0.62 [0.52-0.76]*	0.85 [0.74 - 0.91]*
Low physical activity	1.96 [0.68-5.6]	1.10 [0.36-3.22]	1.81 [0.75 - 4.39]
High coffee consumption	1.76 [1.08-2.85]*	0.85 [0.46-1.57]	0.82 [0.74-0.91]*

BMI: body mass index. *indicates p < 0.05

## Discussion

Low BMD is well-known to be an important risk factor for fractures in older men. In this population of Moroccan males, ageing, a lower BMI, and low physical activity were independently associated to the presence of densitometric osteoporosis.

The effect of age on BMD is consistent across the published studies even if there are some differences according to the measured site[14,15]. At the hip, there is consistent evidence that BMD declines with increasing age. At the lumbar spine, several studies reported that BMD may increase with age (approximately 1.5-3.5% per decade for men aged 60 years and older) whereas other studies reported that lumbar spine BMD decreases with age as it was the case in our study (2.7% per decade in average)[8,16-18]. The loss of bone mass observed with age is due to increased osteoclast-mediated bone resorption, endocortical thinning, and increased cortical porosity that is not offset by new bone formation, resulting in a net loss of bone.

Our observations in terms of BMI are very similar to those of most studies showing that lower BMI scores were associated with BMD loss. However, in our study this association was stronger with hip BMD than with lumbar spine. The magnitude of this relationship is consistent across studies in different geographic regions (China[19], United States[18], Europe [20], Morocco[13]): BMD was approximately 3-7% higher at the hip and lumbar spine for every 10 kg increase in weight.

The Osteoporosis Self-Assessment Tool (OST), which uses a person's age and weight to develop a risk score ([(weight in kilograms - age in years) × 0.2], truncated to an integer), is a simple test that has been evaluated primarily in Asian women. More recently, many evaluations have been performed in men[21-23] and showed that it is reasonably cost-effective to risk-stratify patients who should have a BMD testing.

As study duration varies as well as the accuracy and method of measuring the exercise variable, it is difficult to compare the published studies evaluating the impact of physical activity on BMD. While some studies found a positive and weak association between BMD and physical activity (regular activity or lifetime activity) at both sites[18,24-26], in other studies, physical activity was not independently associated with BMD[19,27,28].

The impact of calcium intake on BMD is also controversial. Some studies found a positive association between calcium intake (dietary and/or supplements) and BMD[18,26,29-31] whereas others did not find an independent association at either BMD sites[19,24,25,28].

Effect of smoking on BMD is also controversial. In our study, only 15% were smokers, so no definitive conclusions can be drawn. In some studies[19,27,28,32], both current and former smokers were at greater risk for low BMD compared with nonsmokers. In these studies, current smokers had greater risk than former smokers at the hip. At the lumbar spine, the risk was similar for current and former smokers[19,27,28,33]. A dose-response relationship with lifetime tobacco exposure (pack-years of smoking) was noted particularly in those who smoked in early adulthood[32].

The relationship between alcohol use and osteoporosis remains unclear. In our study, no one from the population study declared alcohol consumption. Some studies found a positive association between moderate alcohol consumption and BMD at the hip[18,26,34] and/or lumbar spine[18,30,35], while others did not find an independent association at either BMD site[19,24,25].

The effect of coffee consumption has been rarely evaluated in the literature. High coffee consumption was found to be independently associated to lumbar spine osteoporosis in our study. We do not have any clear explanation to that finding. While ingestion of caffeine does produce an immediate increase in urinary calcium loss, this is followed by reduced renal calcium clearance, with no net effect overall. There is also a small impairment of calcium absorption by caffeine, but in the presence of adequate dietary calcium, this effect is thought not to be relevant to overall bone health[36].

History of previous fracture was associated with lower BMD in many studies[26,28]. Even there was a trend in the association between low BMD and history of previous fractures in our study, it may have lacked power to reach the threshold significance.

There are some limitations to our study. The population is composed of volunteers. The study sample was not population-based but recruited from men who were invited by their general practitioner or contacted by word of mouth or through advertisements. We did not examine use of glucocorticoids or any disease states as our goal was to examine healthy men. It is possible that this may introduce a selection bias focusing on the wealthier and better-educated part of the population or alternatively on those who through lifestyle or medical history are more prone to osteoporosis. However, this research did not intend to establish the prevalence of osteoporosis but to identify associations with low bone mass. In the other hand, our study is the first to report risk factors of osteoporosis in men in a region from the south bank of the Mediterranean Sea while all of the published studies in the literature come from American, European and Asian countries.

In conclusion, we have demonstrated that in this population of healthy Moroccan males, ageing and a lower BMI were associated with osteoporosis. Further longitudinal studies are still required to investigate the predictive value of potential factors for osteoporosis and fractures in Moroccan men.

## Competing interests

The authors declare that they have no competing interests.

## Authors' contributions

AEM conceived the design and coordinated the study, participated to the statistical analysis and wrote the manuscript. MG, SG, IG, AM, AR participated in data acquisition. MD participated in the design of the study and performed the statistical analysis. All authors read and approved the final manuscript.

## Pre-publication history

The pre-publication history for this paper can be accessed here:

http://www.biomedcentral.com/1471-2474/11/148/prepub
